# Effectiveness of Low-Frequency Stimulation in Proprioceptive Neuromuscular Facilitation Techniques for Post Ankle Sprain Balance and Proprioception in Adults: A Randomized Controlled Trial

**DOI:** 10.1155/2020/9012930

**Published:** 2020-09-23

**Authors:** Khalid A. Alahmari, Paul Silvian, Irshad Ahmad, Ravi Shankar Reddy, Jaya Shanker Tedla, Venkata Nagaraj Kakaraparthi, Kanagaraj Rengaramanujam

**Affiliations:** Department of Medical Rehabilitation Sciences, College of Applied Medical Sciences, King Khalid University, Abha, Saudi Arabia

## Abstract

Stretching is an important part of post ankle sprain rehabilitation, as well as an effective exercise for improving general ankle-joint performance. But the combination of stretching alongside low-frequency stimulation has not yet been extensively studied. Therefore, the purpose of the present randomized controlled trial was to compare the combined effects of low-frequency transcutaneous electrical nerve stimulation (TENS) with proprioceptive neuromuscular facilitation (PNF) on strength, balance, and proprioception among individuals with post ankle sprain. Sixty male subjects with lateral ankle sprain were selected and randomly allocated to three groups: group 1, group 2, and the control group (CG). Subjects in group 1 received the PNF stretching technique combined with TENS. TENS stimulation was provided using two electrodes placed 5 cm apart directly on the triceps sural muscle of the affected leg and a biphasic current with a symmetrical waveform at 50 Hz for 15 seconds, tuned for a 3-second ramp up time and a 30-second rest time with a 250-microsecond pulse duration was given with PNF stretching. Subjects in group 2 received the PNF stretching technique alone. Both group 1 and group 2 received these treatments for 4 weeks (4 days/week); follow-up assessments were administered in the third and fifth weeks. CG received no treatment; outcome measures alone were assessed. Outcome measures comprised pain, balance, flexibility, proprioception, range of motion, muscle strength, and functional limitation. A mixed-model ANOVA showed significant interaction (time and group) and the time effect for all the outcome measures (*p* ≤ 0.05). Group 1 (PNF-TENS) showed significant improvement for all the outcome variables compared to the other groups. The present study showed PNF stretching combined with TENS for the triceps sural muscle to trigger muscle contraction during the muscle contraction phase of the PNF stretch, compared against PNF stretching alone, produced significant improvements in ankle function for post ankle sprain subjects.

## 1. Introduction

The ankle joint plays a vital role in collecting sensory feedback as well as in controlling balance and posture [1]. Ankle sprain refers to a ligament tear in the ankle, and ligament sprains most commonly occur on the lateral side of the ankle in isolation [2]. Ankle sprains are easily diagnosed [3] because the pain, tenderness, and swelling are usually localized on the outside of the ankle for a patient who has twisted their ankle with inversion [4].

Ankle sprain not only causes damage to the structure of the ligament but also damages the surrounding structures such as the muscles, tendons, and nerves in the ankle complex. Any such injury may lead to ligament laxity of the ankle joint, muscular weakness, and deficits. This injury leads to impairment of joint proprioception, balance, firing of ankle muscles, nerve conduction velocity, cutaneous sensation, and muscle power, as well as restriction of the range of motion of the ankle, especially dorsiflexion [5]. Rehabilitation of ankle injuries requires specific activities and exercises to improve and recover normal function of muscles and ligaments. The journey towards recovering normal function after the rehabilitation phase of ankle post sprain is challenging [6].

The effectiveness of a rehabilitation program after injury or surgery often determines the success of future function and performance [7]. For example, the range of motion and muscle power should return to normal preinjury levels such that functional activities may be performed normally as per preinjury [6]. Most patients with ankle sprain recover completely, but a minority of patients report consistent pain, fear of recurrence, and functional limitation. Intervention to curb and permanently recover from an ankle sprain is still much debated and is coupled with a lack of evidence for the effectiveness of treatment designed to build confidence for patients with chronic ankle sprain [8].

In rehabilitation, the complete care of ankle injuries must include pain management, regaining full ankle range of motion, as well as improving muscle strength, proprioception, and balance [9]. These goals can be achieved by modalities that include flexibility and strengthening exercises, proprioception, and balance training. A structured program of intervention, allowing for the significant effect of time and treatment, is essential for understanding ankle rehabilitation.

Stretching is used in various therapeutic procedures that are designed to increase the length of soft tissue structures that have been shortened due to pathological causes, thereby increasing the range of motion [10]. Tight muscles of the leg are passively stretched, isometrically activated, and then further stretched to increase the ankle range of motion [11].

Proprioceptive neuromuscular facilitation (PNF) is a stretch training technique used to increase flexibility [12]. PNF uses static stretching in combination with triggering isometric muscle contraction. PNF stretch uses muscle contraction to trigger neuromuscular activity, initiate a greater stretch, and increase range of motion [13]. PNF techniques such as the “contract-relax” technique or the “hold-relax” technique can be used to achieve a range of motion (ROM) increase beyond that of traditional stretching. The hold-relax PNF technique is done using agonist contraction, initial stretching, and then isometric contraction of a muscle which is tight followed by concentric contraction to the opposite tight muscle. Hold-relax uses dynamic stretching along with the static stretches isometrically [14, 15].

The widespread knowledge about transcutaneous electrical nerve stimulation (TENS) is mainly to reduce pain and improve function in different painful conditions over the last few years and one of the primary clinical tools for managing pain [16, 17]. Because pain and restriction in the range of motion are commonly reported, the usage of TENS is shown to relieve pain in clinical practice, especially when applied before stretching and therapeutic exercises [18, 19].

It is also reported that in animals and clinical research conducted recently, TENS improved balance, muscle strength, and spasticity [20–22]. TENS is effective in relieving muscle fatigue; muscle fatigue is considered an important factor for voluntary muscle control, posture, and balance [23]. Treatment using TENS reduced knee pain effectively by increasing the quadriceps motor neuron pool and triggering the isometric quadriceps muscle activity [24]. However, the use of this TENS to trigger a muscle contraction during the phase of a muscle contraction during PNF (hold-relax) stretch has not been investigated in previous studies.

Therefore, we hypothesized that TENS application could improve and aid the stretching effect in a synergistic manner thereby increasing the range, proprioception, balance, and flexibility of the muscles. The purpose of this research was to compare pre-, post-, and follow-up effects between the PNF stretching technique combined with TENS and PNF stretching alone for post ankle sprain individuals. A second aim is to determine the treatment effect on pain, balance, flexibility, proprioception, range of motion, muscle strength, and functional limitation between the groups.

## 2. Materials and Methods

The clinical trial was approved by the Institutional Ethics Committee (ECM#2019-26) of King Khalid University, Saudi Arabia. A clinical trial was also registered in the Clinical Trials Registry—ISRCTN 18013941 (UK).

### 2.1. Participants

**Figure 1:**
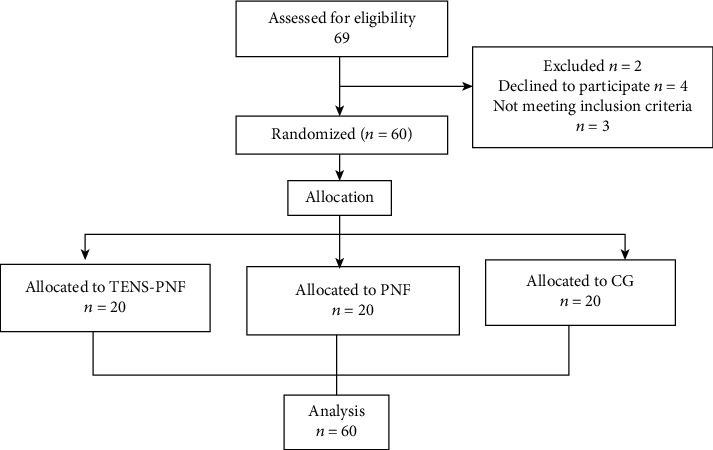
Flow chart enrolment for the study.

After obtaining written informed consent, 69 subjects were screened, consisting of both university students and staff. Of the initial group, 60 subjects had a unilateral lateral ankle sprain and were included in the study as shown in the flow chart (Figure 1), based on the following inclusion criteria: males who sprained their ankle at least 3 months before, aging between 18 and 40 years, who are unable to bend their foot upwards on the post sprain ankle as much as on the normal ankle, and who have been diagnosed by an orthopedic surgeon. Excluded were subjects with general health issues, ankle fracture, dislocation, grade 3 ankle sprains, bony limitation, swelling, neuropathies, or any other neuromuscular pathologies.

### 2.2. Design

The study was a single-blind randomized controlled trial. Subjects were randomly allocated to three groups using block randomization, each group with 20 subjects of the 3 blocks. Concealed allocation was achieved using a computer-generated table of block-randomized numbers. The random numbers were placed in sealed envelopes. The researcher opened the envelopes and proceeded with treatment according to the group assigned. Twenty subjects were randomly allocated to group 1 (PNF-TENS), group 2 (PNF), and the control group (CG).

Group 1 received PNF (hold-relax technique) along with TENS; group 2 received PNF stretching (hold-relax technique) only; and the CG received no treatment. In all, there were 12 treatment sessions, conducted four times per week, for three weeks, in the university clinic. All three groups were assessed at pretreatment, posttreatment in the third week, and follow-up which was recorded in the beginning of the fifth week. Outcome measures were tested by an independent evaluator not involved in providing treatment to subjects as well as allocation. The outcome measures were pain, balance, flexibility, proprioception, range of motion, muscle strength, and functional limitation. The outcomes were measured in the same order throughout the study, prior intervention, at the 3^rd^ week, and at the 5^th^ week.

### 2.3. Outcome Measures

#### 2.3.1. Pain

The visual analog scale (VAS) was recorded using a handwritten mark on a 10 cm line representing a continuum from “no pain” to “worst pain” [25].

#### 2.3.2. Star Excursion Balance Test (SEBT)

The balance was assessed using SEBT and was measured with the subjects standing barefoot at the center of a grid with eight lines extending at 45° angles. Subjects were instructed to touch the farthest point on the line with their distal part of the foot while maintaining the posture. Each subject maintained a single-leg stance and used the contralateral leg to touch as far as possible along the chosen line. The examiner marked the point touched by the foot (distance in cm) from the center of the grid to the point touched by the big toe. Subjects then returned to a bilateral stance and maintained equilibrium. Leg length was measured while subjects were in the supine position, from the anterior superior iliac spine to the distal tip of the medial malleolus in order to normalize the reach distance [26].

**Figure 2:**
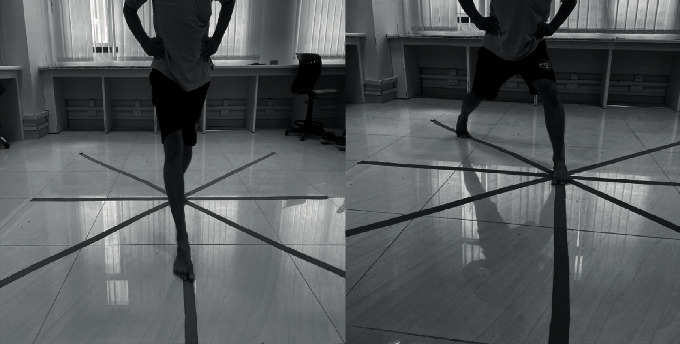
Star Excursion Balance Test in anterior and posterolateral directions.

A valid trial was measured in the same standing posture, and when any change was detected, the subject's stance foot was repositioned to the center of the grid prior to beginning the next trial. The eight lines—anterior (A), posterior (P), medial (M), lateral (L), anterolateral (AL), anteromedial (AM), posterolateral (PL), and posteromedial (PM)—were constructed based on the direction shown in Figure 2. Reach direction order was designed using a Latin square to avoid any order sequence effect that might contaminate the data [27]. Subjects performed two practice trials in each direction with a ten-second rest break between reach trials [28]. After evaluating the primary and secondary outcome measures, the subjects were provided with treatment as per their group protocol.

#### 2.3.3. Flexibility (Knee to Wall Test)

Subjects were asked to stand facing a wall with about 10 cm between their toes and the wall. Subjects were then asked to step back a distance of one foot behind the other foot. The knee was bent to the front until it touched the wall, and the subject was asked to keep that heel in full contact with the floor. If the knee could not touch the wall without the heel coming off the floor, the front foot was moved closer to the wall. This exercise was then repeated, and the distance from the tip of the big toe to the wall was measured [29].

#### 2.3.4. Ankle Proprioception

**Figure 3:**
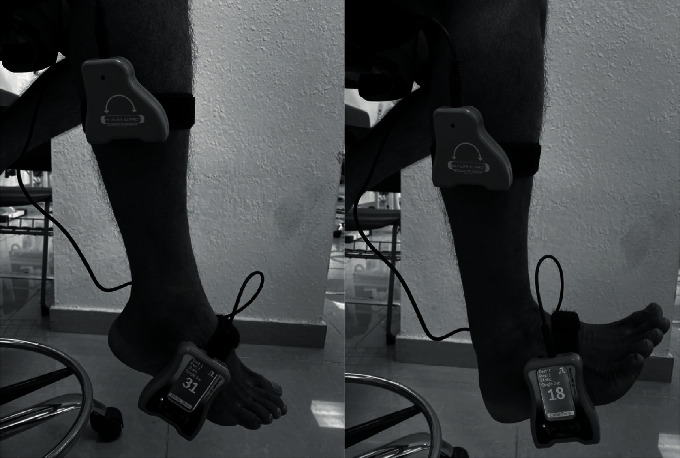
Proprioception of the ankle in plantarflexion and dorsiflexion.

A digital dual inclinometer (Dualer IQ PRO Digital Inclinometer, J-TECH, Midvale, UT 84047, USA) was used to measure joint proprioception of the ankle. Subjects were asked to sit in a high sitting position with their eyes closed. A dual inclinometer was strapped to the midshaft lateral face of the tibia, and the display unit was strapped to the middle of the third lateral border of the foot. The foot was brought to the targeted angle dorsiflexion, and the subject was asked to maintain the position for 10 seconds (in order to remember this position) and then to return to the neutral position. The subject was then asked to bring the foot actively to the target angle once again [30, 31], as shown in Figure 3. The measurement was taken during three consecutive trials from both angles separately (dorsiflexion and plantar flexion). Recorded mean values (in degrees) were used for the analysis of both target positions. The error angle deviation from the target position set angle was used as the result value [32].

#### 2.3.5. Range of Motion (ROM)

The ROM was assessed using a flexi-plastic baseline (USA) standard universal goniometer for measuring dorsiflexion ROM and plantarflexion ROM. Subjects were seated in the high sitting position, with the fulcrum centered over the lateral malleolus of the ankle and the stationary arm parallel to the fibula and tibia. The movable arm of the goniometer followed a line parallel to the 5th metatarsal of the foot. Subjects were asked to dorsiflex and then plantarflex their ankles from the foot-relaxed starting position (considered to be the zero-neutral position), and the average of the three trials was recorded [33].

#### 2.3.6. Muscle Strength

Isometric muscle strength was measured using a strength dynamometer (Baseline, USA). Each participant was positioned in the supine position with their feet over the edge of a plinth. The strength dynamometer was positioned against the metatarsal heads on the plantar surface of the foot to measure the strength of the plantar flexors. The strength dynamometer was positioned on the dorsal aspect of the foot proximal to the metatarsal heads to measure the strength of the dorsiflexors. Each participant performed two practice trials in order to familiarize themselves with the movements prior to testing. Three repetitions were performed for both dorsi- and plantar flexors, with a minimum rest period of 10 seconds between contractions. A single examiner performed all tests for each individual [34].

#### 2.3.7. The Foot and Ankle Disability Index (FADI) Score

Functional limitation was assessed using FADI; all subjects completed the FADI during three different sessions (pre, week 3, and week 5). The completed survey indicated the function of the injured ankle at each session. Each item was scored from 0 (unable to do) to 4 (no difficulty at all), based on 22 questions related to functional activities and four questions related to pain. The FADI has a total of 104 points and is scored as percentages. A total of 100% indicates no dysfunction at all [35].

### 2.4. Intervention

#### 2.4.1. PNF Stretching Method

The PNF technique (hold-relax) was applied for the triceps sural muscle using agonist contraction, after stretching followed by isometric contraction of the tight muscle and followed by concentric contraction to the opposite tight muscle [36]. The hold-relax PNF protocol was performed, with each subject lying prone on a plinth and the therapist resisting the subject's plantar flexion. The therapist followed the fundamental principles of the PNF method in terms of manual contact, body position and body mechanics, verbal commands, and vision [37]. The subject was asked to perform isometric triceps sural muscle contraction for 20 seconds, after which the therapist waited for four seconds before resuming the triceps sural muscle stretch, slowly and continuously, until the subject reported strong but tolerable discomfort and began to feel a stretching sensation. Once this benchmark had been reached, the stretch was maintained for approximately 30 seconds longer. This method was adopted from the previously published work by Esnault and Viel [38] and was performed four times per session on the affected lower limb.

#### 2.4.2. Group 1: TENS-PNF

**Figure 4:**
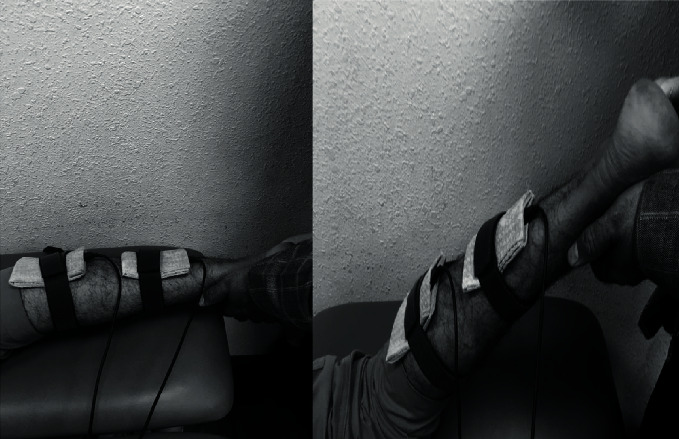
Application of TENS electrode placements used to activate the triceps sural muscle and simultaneous PNF stretching.

This group received PNF stretching, as described above, in the PNF stretching method combined with TENS (Trans Med from Enraf Nonius). Two electrodes (4 × 8 cm) were used for this procedure. The electrodes were placed in water-soaked sponge pouches and strapped on the triceps sural muscle: one was placed 5 cm distal to the popliteal fossa, and the other was placed 5 cm distal to the proximal electrode, directly on the triceps sural muscle of the affected leg as shown in Figure 4. The TENS device unit was adjusted to deliver a biphasic current with a symmetrical waveform at 50 Hz for 15 seconds, tuned for a 3-second ramp up time, and a 30-second rest time with a 250-microsecond pulse duration. The intensity was set to the maximum tolerance limit by each subject and was performed four times per session on the affected lower limb. This method was adopted from the previously published work by Pérez-Bellmunt et al. [39]. Each subject underwent this modified PNF stretching procedure four times per session on the affected lower limb, and the total intervention was 30 minutes. This combination method was applied to achieve stronger isometric contraction.

#### 2.4.3. Group 2: PNF

For this group, the total intervention was 30 minutes and involved PNF stretching alone. The PNF stretching protocol was carried out as previously described in the PNF stretching method.

#### 2.4.4. Group 3: Control Group (CG)

Assessments alone were administered to the control group.

### 2.5. Sample Size Calculation

For the calculation of the minimum sample size, a priori power analysis was performed using G Power 3.1.9.4 software. Prior randomized trials [40, 41] have estimated effect sizes (0.22–0.57) for changes in plantar flexor strength among individuals with post ankle sprain. To generate the current sample size estimate, we used an effect size of 0.22 with Cohen's *d*, an alpha of 0.05, and power of 90%. A sample size of 20 subjects in each group was used to detect a time × group interaction.

### 2.6. Statistical Analysis

The normality of distribution of all variables was verified using the Shapiro-Wilk test (*p* ≤ 0.05). The three group's baseline and demographic characteristics were compared using one-way ANOVA for parametric variables and chi-square test for the nonparametric variables. Levene's output was used to check the homogeneity between the groups. A mixed-model ANOVA was used to see the time effect, group effect, and time × group interaction effect between the three groups. Mauchly's test was used to test assumptions of sphericity; since the degrees of freedom were violated, it was rectified by using the Greenhouse-Geisser estimates of sphericity. The partial eta square *η*_*p*_^2^ was obtained from Greenhouse-Geisser within the subject effect. Statistical significance was indicated at *p* ≤ 0.05, and the confidence interval was set at 95%. Statistical Package for the Social Sciences version 22.0 (IBM Corp., Armonk, NY, USA) was used for all the analysis.

## 3. Results

Pooled means and standard deviations of all outcome variables as well as *p* values showed no significant difference, except for the SEBT in the anterior direction, as shown in Table 1. Levene's output showed no significant differences between the three groups, indicating homogeneity between the groups. Mixed-model ANOVA revealed a significant time effect and time × group interaction effect (*p* ≤ 0.01) for pain, flexibility, proprioception, ROM, muscle strength, FADI score, and balance. The group effect was significant in the anterior, posterior, posterolateral, and posteromedial directions for balance (*p* ≤ 0.01). Meanwhile, functional limitation (FADI) scores (*p* ≤ 0.09) and dorsiflexion ROM as well as balance in the medial, lateral, anterolateral, and anteromedial directions, did not reveal significant difference between the groups (*p* ≥ 0.05) as shown in Tables 2 and 3.

**Figure 5:**
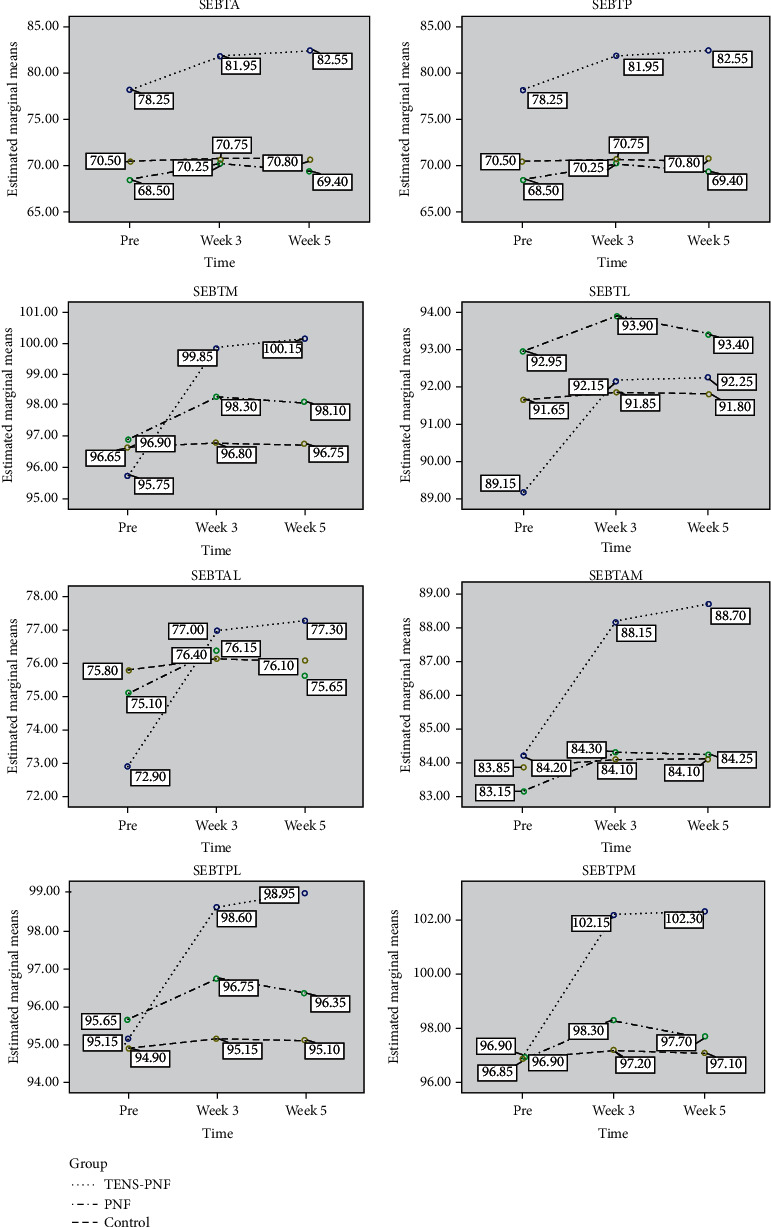
Mean values of the group and time interaction effect of the outcome variables measured at the pre, 3^rd^ week, and 5^th^ week for Star Excursion Balance Test (SEBT). A: anterior; P: posterior; M: medial; L: lateral; AM: anteromedial; AL: anterolateral; PM: posteromedial; PL: posterolateral.

**Figure 6:**
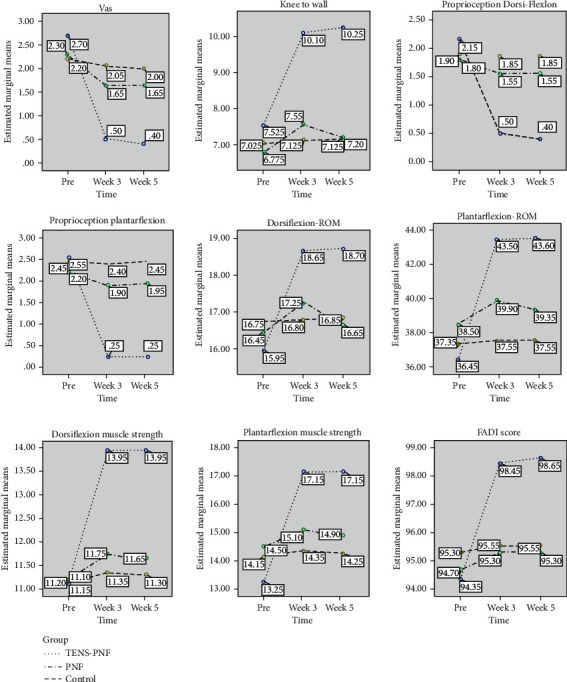
Mean values of the group and time interaction effect of the outcome variables measured at pre, 3^rd^ week, and 5^th^ week for pain, flexibility, proprioception, range of motion, muscle strength, and functional limitation. VAS: Visual Analogue Scale; ROM: range of motion.

The TENS-PNF group in the present study showed significant decrease in pain in the pre- to follow-up period 85.1% compared to PNF 28.2%. The TENS-PNF group showed a significant increase in balance after treatment for anterior, posterior, posterolateral, and posteromedial directions in the pre- to follow-up period (5.4%, 4.2%, 3.9%, and 5.6%) compared to PNF (1.3%, 1.2%, 0.7%, and 0.8%, respectively). The TENS-PNF group showed significant increase in flexibility after treatment in pre- to follow-up was 36.3% compared to PNF 5.9%. The TENS-PNF group also showed a significant increase for proprioception in dorsiflexion after treatment in the pre- to follow-up period (81.3%) compared to PNF (13.8%), and a similar result was seen for proprioception in plantar flexion (89.7%) compared to PNF (11.3%). The TENS-PNF showed significant increase in planter flexion ROM in the pre- to follow-up (19.7%) compared to PNF (2%). The TENS-PNF showed a significant increase in muscle strength for dorsiflexors in the pre to follow-up (25.2%) compared to PNF (4%); a similar increase was also observed for the plantar flexors (29.4%) compared to PNF (2.7%). CG did not show any significant change in time, group, or time × group interaction effect for the variables. There comparison before and after treatment showed a significant difference between the time and measurements, as shown in Figures 5 and 6 for all the variables.

## 4. Discussion

Many studies have reported various types of stretching techniques and methods to address musculoskeletal problems and the impacts those problems have on sports performance and disability testing [42]. Studies reported that a minimum of 2-minute stretching of the gastrocnemius significantly produces an ROM increase and a decrease of stiffness in the muscle-tendon unit [43]. Several reports which indicated stretching conditions and methods of application have also examined time parameters [44]. Furthermore, many protocols based on the published results of previous studies have been found to contradict the results of those studies; therefore, the authors of this study selected a PNF stretching protocol from previously published research [45].

In a study conducted on young anterior knee pain subjects using TENS and PNF technique, it was concluded that pre- to postintervention the pain pressure threshold values decreased corresponding to pain similar to other studies, and also an increase in ROM values proved muscle relaxation and the significant change they observed from pre to 6-minute posttreatment values was in functional vertical jump demonstrating the effect on muscle performances for both the TENS group and the PNF group [46]. Various studies have provided mixed conclusions on the effect of PNF stretching and the permanence of ROM gains. For example, one study reported that ROM improvements were not immediately significant 6 minutes after 5 repetitions of PNF stretching [47].

Another study, however, concluded that even after a single repetition of PNF stretching, ROM was significantly higher than baseline values. This improvement was evident 90 minutes after cessation of intervention for the muscle groups stretched [48]. Several other studies have also noted that ROM increments decrease quite sharply once intervention ceases [47, 49] and therefore recommend that PNF stretching should be conducted at least once or twice weekly. The present study's results show that ROM significantly increased when combining the TENS and PNF intervention 4 times per week for 3 weeks, and the gained ROM did not decline, which was observed in the follow-up assessment. Karasuno et al.'s [18] results support the present study; they concluded that TENS combined with stretching is effective in reducing pain and decreasing the muscle hardness, ultimately increasing the ROM.

TENS causes elicited muscle contractions and also allows for the activation of a greater proportion of type II muscle fibers compared to volitional exercise at comparable intensity [50–52]. Kang et al. [53] suggested that TENS, when applied directly to the skin overlying the gastrocnemius (calf muscle), is effective in improving balance for healthy adults, which concurs with the results of the present study. TENS not only improves strength but also increases joint position sense and balance [54]. The present study used TENS in combination with PNF stretching and found significant improvement in strength, balance, and proprioception compared to PNF and CG.

The results of a randomized controlled trial for children with hamstring syndrome who were given stretching combined with TENS indicated that the said combination produced better results than a protocol without TENS combinations [55]. A similar study also proved that TEN-PNF significantly improved flexibility of the hamstring muscles [39]. Another study applied TENS combined with PNF stretching for the hamstring muscles in volleyball players, following a very similar design [56]. Similar results were found in a study conducted for healthy women to achieve flexibility gains on their hamstrings that found that there was no increase in muscle flexibility compared to the group which did not receive TENS; based on their findings, they concluded that the combination of TENS and stretching decreases the resistance imposed by neurological and viscoelastic properties and will significantly increase muscle flexibility compared to an isolated technique [57]. The present study results also concur with the previous studies, proving that flexibility significantly improved with combination of TEN-PNF.

It can be held that TENS-PNF showed a significant difference and improvement attributable to the autogenic inhibition reflex, which is the reflex produced when a Golgi organ registers an increase in muscle tension. In our case, contraction in the PNF later provokes a reflex relaxation of the muscles [58]. This reflex may be triggered more when using the TENS because of its unique electrical stimulation that can trigger a tetanic contraction, simultaneously improving muscle strength and increasing tension in the Golgi organ [59]. Considering the evidence indicating that TENS could play an important role in stretching programs, the present study contributes to the existing evidence in favor of PNF combined with TENS for the triceps sural muscle, and the results of the present study support these earlier works.

The results of the present randomized controlled trial suggest that the use of TENS low-frequency currents improved the results of PNF stretching when applied directly to the triceps sural muscle and, therefore, that the results obtained with this stretching modality are significant. The study was conducted with male subjects only. A comparison study with females to understand the response to PNF stretching, with and without low-frequency electrical stimulation, should also be examined. The present study was only conducted for subjects participating in recreational sports; results may differ for professional athletes.

## 5. Conclusions

The present study showed that a 12-session treatment program, spread over 3 weeks, triceps sural muscle PNF stretching combined with TENS triggered muscle contraction during the muscle contraction phase of the PNF stretch, compared against PNF stretching, produced significant improvements in balance, proprioception, strength, and range of motion, while also yielding reducing pain for post ankle sprain subjects. It was also demonstrated that the treatment effect was sustained even after cessation of treatment to the follow-up assessment in the 5^th^ week. For this reason, this treatment procedure will likely be helpful in rehabilitating post ankle sprain patients by improving overall function and helping build confidence in capacity for physical activity.

## Figures and Tables

**Table 1:** Data (mean and standard deviation) of variables prior to intervention for each group.

Variables	Group 1 TENS-PNF (*n* = 20)	Group 2 PNF (*n* = 20)	Control group (CG) (*n* = 20)	*p* value
Age (years)	25.8 ± 5.7	25.7 ± 5.6	25.9 ± 6.2	0.991
Height (meters)	1.6 ± 0.0	1.7 ± 0.0	1.7 ± 0.0	0.276
Weight (kg)	65.9 ± 14.1	72.6 ± 15.4	68.9 ± 13.6	0.346
BMI	23.0 ± 5.5	24.5 ± 4.5	23.1 ± 4.2	0.573
Leg length (cm)	83.4 ± 10	85.0 ± 5.4	86.3 ± 6.3	0.483
SEBT anterior	78.2 ± 2.5	68.5 ± 3.9	70.5 ± 5.9	0.01^∗^
SEBT posterior	92.4 ± 3.1	91.2 ± 4.6	90.7 ± 5.4	0.494
SEBT medial	95.7 ± 3.5	96.9 ± 3.7	96.6 ± 3.5	0.576
SEBT lateral	89.1 ± 5.9	92.9 ± 4.7	91.6 ± 5.5	0.090
SEBT anterolateral	74.9 ± 4.7	75.1 ± 3.9	75.8 ± 4.1	0.091
SEBT anteromedial	84.2 ± 5.8	83.1 ± 5.7	83.8 ± 5.7	0.843
SEBT posterolateral	95.1 ± 3.0	95.6 ± 2.9	94.9 ± 2.8	0.711
SEBT posteromedial	96.9 ± 2.6	96.8 ± 2.8	96.9 ± 3.3	0.998
VAS	2.7 ± 1.0	2.3 ± 1.0	2.2 ± 0.9	0.270
KNEE to wall (cm)	7.5 ± 1.8	6.7 ± 0.8	7.0 ± 0.8	0.180
Dorsi proprioception	2.1 ± 1.2	1.8 ± 1.0	1.9 ± 1.0	0.599
Plantar proprioception	2.5 ± 1.7	2.2 ± 1.9	2.4 ± 1.2	0.722
Dorsi ROM	15.9 ± 1.5	16.4 ± 1.8	16.7 ± 1.7	0.336
Plantar ROM	36.4 ± 3.9	38.5 ± 3.3	37.3 ± 4.0	0.224
Dorsi MS (kg)	11.1 ± 1.8	11.2 ± 0.8	11.1 ± 0.8	0.971
Plantar MS (kg)	13.2 ± 1.7	14.5 ± 0.9	14.1 ± 1.4	0.020
FADI score	94.3 ± 2.5	94.7 ± 3.5	95.3 ± 3.8	0.665

VAS: Visual Analogue Scale; BMI: Body Mass Index; ROM: range of motion; MS: muscle strength. ^∗^Significant (*p* ≤ 0.01).

**Table 2:** Summary of statistical results of the mixed ANOVA and pooled means and standard deviations of all variable measures.

Variables	Group 1 (TENS-PNF) Group 2 (PNF) Control group (CG)	Mean (standard deviation)			Mixed ANOVA (*p* value)		
		Pre (1)	3^rd^ week (2)	5^th^ week (3)	Interaction (group and time) effect *η*_*p*_^2^ (*p* value)	Group (*G*) effect *η*_*p*_^2^ (*p* value)	Time (*T*) effect *η*_*p*_^2^ (*p* value)
Pain VAS (cm)	TENS-PNF	2.7 ± 1.0	0.5 ± 0.6	0.4 ± 0.5	0.608 (0.01)^∗^	0.192 (0.002)^∗^	0.674 (0.01)^∗^
	PNF	2.3 ± 1.0	1.6 ± 0.8	1.6 ± 0.8			
	CG	2.2 ± 0.9	2.0 ± 0.8	2.0 ± 0.8			
Knee to wall (cm)	TENS-PNF	7.5 ± 1.8	10.1 ± 1.2	10.2 ± 1.2	0.661 (0.01)^∗^	0.482 (0.01)^∗^	0.661 (0.01)^∗^
	PNF	6.7 ± 0.8	7.5 ± 0.9	7.2 ± 1.0			
	CG	7.0 ± 0.8	7.1 ± 0.8	7.1 ± 0.8			
Dorsi proprioception	TENS-PNF	2.1 ± 1.2	0.5 ± 0.5	0.4 ± 0.5	0.438 (0.01)^∗^	0.141 (0.01)^∗^	0.390 (0.01)^∗^
	PNF	1.8 ± 1.0	1.5 ± 1.0	1.5 ± 1.0			
	CG	1.9 ± 1.0	1.8 ± 1.0	1.8 ± 1.0			
Plantar proprioception	EG 1	2.5 ± 1.7	0.2 ± 0.5	0.2 ± 0.5	0.520 (0.01)^∗^	0.244 (0.01)^∗^	0.439 (0.01)^∗^
	PNF	2.2 ± 1.9	1.9 ± 1.0	1.9 ± 1.1			
	CG	2.4 ± 1.2	2.4 ± 1.3	2.4 ± 1.2			
Dorsiflexion ROM	TENS-PNF	15.9 ± 1.5	18.6 ± 1.8	18.7 ± 1.8	0.655 (0.01)^∗^	0.064 (0.15)^#^	0.622 (0.01)^∗^
	PNF	16.4 ± 1.8	17.2 ± 1.9	18.7 ± 1.8			
	CG	16.7 ± 1.7	16.8 ± 1.9	16.8 ± 1.8			
Plantarflexion ROM	TENS-PNF	36.4 ± 3.9	43.5 ± 3.7	43.6 ± 3.9	0.729 (0.01)^∗^	0.148 (0.01)^∗^	0.693 (0.01)^∗^
	PNF	38.5 ± 3.3	39.9 ± 3.4	39.3 ± 3.0			
	CG	37.3 ± 4.0	37.5 ± 4.1	37.5 ± 4.3			
Dorsiflexion MS	TENS-PNF	11.1 ± 1.8	13.9 ± 1.7	13.9 ± 1.7	0.741 (0.01)^∗^	0.298 (0.01)^∗^	0.736 (0.01)^∗^
	PNF	11.2 ± 0.8	11.7 ± 0.7	11.6 ± 0.6			
	CG	11.1 ± 0.8	11.3 ± 0.8	11.3 ± 0.8			
Plantarflexion MS	TENS-PNF	13.2 ± 1.7	17.1 ± 1.7	17.1 ± 1.7	0.594 (0.01)^∗^	0.218 (0.01)^∗^	0.541 (0.01)^∗^
	PNF	14.5 ± 0.9	15.1 ± 0.9	14.9 ± 1.0			
	CG	14.1 ± 1.4	14.3 ± 1.5	14.2 ± 1.5			
FADI score	TENS-PNF	94.3 ± 2.5	98.4 ± 1.9	98.6 ± 1.7	0.678 (0.01)^∗^	0.078 (0.098)^#^	0.651 (0.01)^∗^
	PNF	4.7 ± 3.5	5.3 ± 3.4	95.3 ± 3.4			
	CG	5.3 ± 3.8	5.5 ± 3.7	95.5 ± 3.7			

VAS: Visual Analogue Scale; ROM: range of motion; MS: muscle strength; FADI: the Foot and Ankle Disability Index score. ^∗^Significant effect (*p* ≤ 0.05). ^#^Nonsignificant.

**Table 3:** Summary of statistical results of the mixed ANOVA and means and standard deviations of balance.

Balance	Group 1 (TENS-PNF) Group 2 (PNF) Control group (CG)	Mean (standard deviation)			Mixed ANOVA (*p* value)		
		Pre	3^rd^ week	5^th^ week	Interaction (group and time) effect *η*_*p*_^2^ (*p* value)	Group (*G*) effect *η*_*p*_^2^ (*p* value)	Time (*T*) effect *η*_*p*_^2^ (*p* value)
SEBT anterior	TENS-PNF	78.2 ± 2.5	81.9 ± 3.1	82.5 ± 2.6	0.613 (0.01)^∗^	0.593 (0.01)^∗^	0.670 (0.01)^∗^
	PNF	68.5 ± 3.9	70.2 ± 4.1	69.4 ± 4.2			
	CG	70.5 ± 5.9	70.7 ± 7.0	70.8 ± 7.3			
SEBT posterior	TENS-PNF	92.4 ± 3.1	96.1 ± 3.1	96.3 ± 3.1	0.655 (0.01)^∗^	0.136 (0.01)^∗^	0.710 (0.01)^∗^
	PNF	91.2 ± 4.6	92.4 ± 4.5	92.3 ± 4.7			
	CG	90.7 ± 5.4	90.9 ± 5.3	91.0 ± 5.2			
SEBT medial	TENS-PNF	95.7 ± 3.5	99.8 ± 4.1	100.1 ± 4.1	0.727 (0.01)^∗^	0.043 (0.28)^#^	0.758 (0.01)^∗^
	PNF	96.9 ± 3.7	98.3 ± 3.5	98.1 ± 3.7			
	CG	96.6 ± 3.5	96.8 ± 3.3	96.7 ± 3.4			
SEBT lateral	TENS-PNF	89.1 ± 5.9	92.1 ± 5.9	92.2 ± 5.7	0.607 (0.01)^∗^	0.033 (0.38)^#^	0.624 (0.01)^∗^
	PNF	92.9 ± 4.7	93.9 ± 4.2	93.4 ± 4.1			
	CG	91.6 ± 5.5	91.8 ± 5.4	91.8 ± 5.1			
SEBT anterolateral	TENS-PNF	74.9 ± 4.7	77.0 ± 4.6	77.3 ± 4.7	0.653 (0.01)^∗^	0.001 (0.96)^#^	0.671 (0.01)^∗^
	PNF	75.1 ± 3.9	76.4 ± 3.8	75.6 ± 4.0			
	CG	75.8 ± 4.1	76.1 ± 4.0	76.1 ± 4.0			
SEBT anteromedial	TENS-PNF	84.2 ± 5.8	88.1 ± 5.9	88.7 ± 5.8	0.562 (0.01)^∗^	0.062 (0.15)^#^	0.602 (0.01)^∗^
	PNF	83.1 ± 5.7	84.3 ± 5.7	84.2 ± 5.4			
	CG	83.8 ± 5.7	84.1 ± 5.8	84.1 ± 5.8			
SEBT posterolateral	TENS-PNF	95.1 ± 3.0	98.6 ± 2.4	98.9 ± 2.4	0.678 (0.01)^∗^	0.118 (0.02)^∗^	0.703 (0.01)^∗^
	PNF	95.6 ± 2.9	96.7 ± 3.2	96.3 ± 3.4			
	CG	94.9 ± 2.8	95.1 ± 2.9	95.1 ± 2.9			
SEBT posteromedial	TENS-PNF	96.9 ± 2.6	102.1 ± 4.0	102.3 ± 4.0	0.633 (0.01)^∗^	0.181 (0.01)^∗^	0.638 (0.01)^∗^
	PNF	96.8 ± 2.8	98.3 ± 3.2	97.7 ± 3.3			
	CG	96.9 ± 3.3	97.2 ± 3.0	97.1 ± 3.0			

SEBT: Star Excursion Balance Test. ^∗^Significant effect (*p* ≤ 0.05). ^#^Nonsignificant.

## Data Availability

The datasets used and/or analyzed during the current study are available from the corresponding author on reasonable request.
